# Is human papillomavirus involved in laryngeal neuroendocrine carcinoma?

**DOI:** 10.1007/s00405-012-2075-7

**Published:** 2012-06-15

**Authors:** Gyorgy B. Halmos, Tom P. van der Laan, Bettien M. van Hemel, Frederik G. Dikkers, Lorian Slagter-Menkema, Bernard F. A. M. van der Laan, Ed Schuuring

**Affiliations:** 1 Department of Otolaryngology, Head and Neck Surgery, University Medical Center Groningen, University of Groningen, P.O. Box 30.001, 9700 RB Groningen, The Netherlands; 2 Department of Pathology, University Medical Center Groningen, University of Groningen, Groningen, The Netherlands

**Keywords:** Human papillomavirus, Immunohistochemistry, Laryngeal neuroendocrine carcinoma, Prognosis

## Abstract

The purpose of this study was to detect human papillomavirus (HPV) infection in laryngeal neuroendocrine carcinoma (LNEC) and to explore the possible relationship between HPV-induced malignant transformation and prognosis in LNEC. Ten cases of LNEC from a tertiary referral hospital were retrospectively analyzed. Clinical data were subtracted from patients’ files. Pretreatment biopsy material was tested for the presence of HPV6, 11, 16, and 18 using a PCR-based detection method. Immunohistochemical staining was performed for Ki-67, p16^INK4A^, and p53 expression. All cases were negative for the low-risk HPV types HPV6 and HPV11 that are associated with laryngeal papillomatosis. High-risk HPV was detected in two cases; an atypical carcinoid was positive for HPV16 and a large-cell neuroendocrine carcinoma for HPV18. Both HPV-positive tumors had a high Ki-67 labeling index. Two of the four cases with a good response to therapy were hrHPV-positive (both HPV DNA positive) compared with none of the five poor responders. Our findings show that HPV may play a role in the pathogenesis of LNEC. The relationship between HPV, improved prognosis and good response to therapy for squamous cell carcinoma of the head and neck may also be true for a subset of LNEC.

## Introduction

Human papillomavirus (HPV) is well known for its involvement in the carcinogenesis of cervical cancer with an estimated prevalence of almost 100 % and mainly concerns high-risk HPV types (hrHPV) including HPV16, 18, 31, 33, 45 [1]. HrHPV has also been implicated in oropharyngeal [2, 3] and laryngeal cancer [4]. The presence of hrHPV in head and neck squamous cell carcinoma (HNSCC) was reported to be associated with a good response to radiotherapy [5, 6]. For oropharyngeal cancer in particular, a strong correlation has been established between the response to therapy and the presence of hrHPV in tumor tissue [7]. This association has led to an increased interest in the relationship between HPV and other tumors of the head and neck region.

A classical site in the head and neck area affected by HPV is the larynx. Most cases with laryngeal papillomatosis are associated with low-risk HPV (lrHPV) types HPV6 and HPV11. In a recent review [4], it is concluded that the role of hrHPV infection in laryngeal squamous cell carcinoma (LSCC) is not yet well established, with several larger studies reporting heterogeneous results. The reported presence of hrHPV in LSCC tumor tissue varied between 7.4 and 58.8 %. No reliable data are available concerning the prevalence of HPV in normal laryngeal tissue, but estimates run as high as 19 % [4].

Although rare, laryngeal neuroendocrine carcinoma (LNEC) are the second most common group of cancers of the larynx, after LSCC. These neoplasms form a rare group of tumors with divergent clinical behavior and prognosis [8, 9]. Little is known about the association of HPV with LNEC. A relationship between HPV and neuroendocrine carcinoma of the cervix has already been established [10, 11]. Presently, there is only one case report that tested the tumor tissue of a patient with a LNEC for HPV, with negative result [12].

The goal of this study was to evaluate the possible involvement of hrHPV and lrHPV in LNEC. Since hrHPV encodes for the oncogenic E6 and E7 proteins inactivating p53 and pRB, respectively, resulting in increased proliferation of tumor cells [1], we also performed immunohistochemistry for Ki-67, p53, and p16. For this purpose, we selected all LNEC cases treated in our Institute between 1988 and 2010.

## Materials and methods

### Patients

The Dutch nationwide digital database of histo- and cytopathology (PALGA) was queried for LNEC diagnosed at the Department of Pathology of the University Medical Center Groningen (UMCG) between 1988 and 2010. All pathology reports were revised by an experienced head and neck pathologist.

Corresponding clinical data concerning age, gender, smoking history, tumor stage, treatment, disease-free survival, recurrence, salvage treatment, and overall and disease-specific survival were collected from the medical charts and electronic patient dossiers of the Department of Otolaryngology, Head and Neck Surgery of the same institution. Age at onset corresponds with the patients’ age in years at the time of the histopathological diagnosis. Disease-free survival was defined as the time in months from the last day of treatment to the first follow-up date where symptoms of recurrence were apparent. Survival times were calculated in months by subtracting the last day of treatment from the last follow-up date. The study was approved by the Institutional Review Board of the UMCG. Patients gave written informed consent prior to biopsies taken.

### Sample collection procedure, DNA isolation, and HPV detection and typing

A 4-μm section was obtained from each paraffin block and stained with hematoxylin and eosin to confirm the presence of tumor tissue in the primary tumor. Primary tumors with <70 % tumor cells were macrodissected to enrich for tumor cells. DNA was extracted from consecutive formalin-fixed paraffin embedded tissue sections as reported previously [13]. Three 10 μm tissue sections were transferred to eppendorf tubes. DNA was extracted by overnight incubation at 56 °C in 250 μl buffer containing SDS-proteinase K (600 μg/ml), heated to 100 °C for 5 min to inactivate proteinase K, and centrifuged at room temperature at 13,000 rpm. The aqueous solution was transferred into a new eppendorf tube and directly used for PCR analysis or stored at −20 °C. The concentration of DNA was determined using the Nanodrop.

For the detection of high-risk HPV, 100 ng genomic DNA extracted from the paraffin embedded tissue was analyzed by PCR using HPV16- and HPV18-specific primers as described previously [14]. For the detection of the presence of the low-risk HPV6 and HPV11, genomic DNA was analyzed using specific HPV6-PCR-primers (HPV6.1: 5′ TAGTGGGCCTATGGCTCGTC and HPV6.2: 5′ TCCATTAGCCTCCACGGGTG) and HPV11-specific primers (HPV11.1: 5′ GGAATACATGCGCCATGTGG and HPV11.2: 5′ CGAGCAGACGTCCGTCCTCG) as described previously [15]. On all HPV-negative cases, a general primer-mediated PCR using the HPV consensus primer set GP5+/6+ with subsequent nucleotide sequence analysis was used as described previously [14].

As a control for the analytical specificity and sensitivity of each hrHPV-PCR, a serial dilution of DNA extracted from CaSki (ATCC; CRL1550; 500 integrated HPV16 copies), HeLa (ATCC; CCL2; 20–50 integrated HPV18 copies), SiHa (ATCC; HTB35; 1–2 integrated HPV16 copies), CC10B (HPV45-positive cell line) and CC11 (HPV67 positive cell line) [16], and HPV-negative cell lines were included in each experiment. DNA—extracted from HPV6- and HPV11-positive laryngeal papillomas that were previous identified—was used for the analytical specificity of the HPV6- and HPV11 PCR.

All standard precautions were taken to avoid contamination of amplification products using separate laboratories for pre- and post-PCR handling. To avoid cross-contamination, a new microtome blade was used each time a new case was sectioned. For quality control, genomic DNA was amplified in a multiplex PCR containing a control gene primer set resulting in products of 100, 200, 300, 400, and 600 bp according to the BIOMED-2 protocol [17]. Only DNA samples with PCR products of 300 bp and larger were used for the detection of HPV. All samples were tested on DNA extracted from two independent slides (duplicates).

### Immunohistochemistry

Paraffin-embedded formalin-fixed sections (4 μm) were deparaffinized and antigen retrieval was performed by overnight incubation at 80 °C in 0.1 M Tris/HCl buffer pH = 9.0 Ki-67, or heating in a microwave oven for 15 min in 10 mM Tris/1 mM EDTA buffer pH = 9.0 for p53. After blocking endogenous peroxidase with 0.3 % hydrogen peroxide, sections were stained for 1 h at room temperature with an antibody against Ki-67 (mouse monoclonal antibody, clone MIB-1, 1:350, DakoCytomation), or p53 (mouse monoclonal, clone DO-7, 1:1,000, DakoCytomation) diluted in phosphate buffered saline (PBS) containing 1 % bovine serum albumin (BSA). The secondary (rabbit anti-mouse peroxidase) and tertiary (goat anti-rabbit peroxidase) antibodies for Ki-67, or Envision (DAKO) for p53 were precipitated using 3.3 diaminobenzidine tetrachloride as a substrate, and slides were counterstained using hematoxylin. For p16^INK4A^ immunohistochemistry, the CINtec™ Histology kit (MTM Laboratories AG, Germany) was used. Scoring of the immunohistochemical stainings was performed as previously described [18]. For both Ki-67 and p53, the percentage of positive nuclear tumour cells was counted. A cut-off above 70 % was considered positive for p53 and Ki-67. p16^INK4A^ protein expression was scored as positive if there was a strong and diffuse nuclear and cytoplasmic staining present in greater than 70 % of the malignant cells as described previously [19].

## Results

### Clinical data

A total of ten cases of LNEC were retrieved from the pathology-database. Formalin-fixed, paraffin-embedded, pretreatment biopsy material was available for all ten cases. The clinical characteristics and immunohistochemical features of these cases are summarized in Table 1.

**Table 1:** Clinical characteristics, HPV status, and immunohistochemical features of ten cases of laryngeal neuroendocrine carcinoma

Case	Age (years)/sex	Histol. subtype	TNM	Treatment^a^	Recurrence	Salvage^a^	HPV 16	HPV 18	HP GP5+/6+	p16	Ki-67 (labeling index) (%)	p53 expression (%)	Follow-up (months)
1	69/M	AC	T1N0M0	TLE	Regional, distant	MRND	–	–	–	–	1	–	DOD (74)
2	57/M	SCNEC	T2N2cM0	CRX (Etoposide, Carboplatin) (37.5 Gy)	Distant	Palliative chemotherapy (Carboplatin/Paclitaxel)	–	–	–	–	70	1	DOD (11)
3	57/F	TC	T1N0M0	Rx (70 Gy)	–	–	–	–	–	–	1	–	NED (96)
4	51/F	AC	T2N0M0	TLE	Regional	RND	–	Positive	Positive	–	90	–	NED (215)
5	81/M	LCNEC	T4aN2bM0	Rx (70 Gy)	Regional	TLE, MRND	–	–	–	–	10	–	AWD (48)
6	75/F	AC	T2N0M0	MLS + CO_2_-laser resection (supraglottic laryngectomy)	Regional, distant	TLE	–	–	–	–	10	1	AWD (26)
7	73/M	LCNEC	T2N0M0	Rx (70 Gy)	Regional, distant	TLE, Chemotherapy (etoposide)	–	–	–	–	1	–	DOD (30)
8	67/M	AC	T3N0M0	Rx (70 Gy)	Regional	TLE	–	–	–	–	10	<1	NED (27)
9	75/M	AC	T1N0M0	MLS + CO_2_-laser resection (chordectomy)	–	–	–	–	–	Positive	20	30	DOOC (19)
10	53/M	LCNEC	T3N2bM0	TLE, MRND, Radiotherapy (60 Gy)	–	–	Positive	–	Positive	–	70	–	NED (105)

*AC* atypical carcinoid; *SCNEC* small-cell neuroendocrine carcinoma; *TC* typical carcinoid; *LCNEC* large-cell neuroendocrine carcinoma; *TLE* total laryngectomy; *CRX* chemoradiation; *Rx* radiotherapy; *MLS* microlaryngosurgery; *MRND* modified radical neck dissection; *RND* radical neck dissection; *DOD* dead of disease; *NED* no evidence of disease; *AWD* alive with disease; *DOOC* dead of other cause

^a^Sort of chemotherapy or surgery and radiation dose

There were seven male and three female patients. The median age at the time of diagnosis was 68 years (range 51–81). The histological classification of the tumors was atypical carcinoid (AC) in five and large cell neuroendocrine carcinoma (LCNEC) in three. Of the two remaining patients, one presented with a typical carcinoid (TC) and the other with a small-cell neuroendocrine carcinoma (SCNEC). The tumor stage on presentation was stage I, II, III, and IVa in, respectively, three, three, one, and three cases; the TNM classification of each case is shown in Table 1.

Four patients underwent surgery, four received radiotherapy, one was treated with a combination of chemo- and radiotherapy and another with surgery followed by postoperative radiotherapy. Surgery varied from laryngeal preservation techniques using transoral CO_2_ laser surgery (2 cases) to total laryngectomy (TLE) (3 cases). Resection margins were free of tumor cells in all patients who underwent TLE (3 cases). In the two laser-resection specimens radicality could not be evaluated. The patients who received radiotherapy were treated with a median total dose of 64 Gy (range 37.5–70). One patient received concomitant chemotherapy with palliative intention, consisting of a combination of etoposide and carboplatin.

Seven of the ten patients developed loco-regional recurrence. Four patients developed distant metastasis, of which three were cutaneous. Mean time to recurrence was 35 months (95 % CI 9–60). Salvage therapy was performed in all but one case (6/7). This patient had a second primary pulmonary tumor at that time which was deemed irresectable and the patient abstained from further treatment. Four patients were laryngectomized (one in combination with a neck dissection and another one with postoperative chemotherapy) and two patients with only lymph node metastasis underwent neck dissection. Resection margins were free of tumor in all of the patients who underwent salvage surgery. The patient with TC developed loco-regional recurrence. There was no relationship between tumor stage or choice of initial treatment and recurrence.

Two patients died of their disease after a median follow-up of 52 months (range 30–74). One patient died of unrelated pulmonary malignancy. The seven censored patients were followed for a median time of 48 months (range 26–215). At the last follow-up, four were without evidence of disease and three were alive with disease. Mean overall survival and disease-specific survival time was 140 months (95 % CI 74–205) and 155 months (95 % CI 88–222), respectively. Tumor type, stage, location, and or initial treatment were not significantly related to either overall or disease-specific survival.

### HPV typing and immunohistochemistry

Of the ten LNEC, two cases were positive for hrHPV (HPV16 and HPV18, respectively). All other cases were negative for both the hrHPV-consensus GP5+/6+-PCR as well as the specific HPV16 and HPV18 PCR. None of the ten LNEC was positive for the lrHPV types HPV6 or HPV11.

Immunohistochemical staining for p16^INK4A^ was positive in only one (hrHPV-negative) case. The HPV16- and HPV18-positive samples both showed overexpression of Ki-67 versus only one out of eight of the HPV-negative cases. High p53 expression was not found in any of the cases.

### Prognosis of HPV-positive LNEC

The HPV16-positive tumor was interpreted as a LCNEC, staged T3N2bM0 and treated with surgery and radiotherapy. The patient is alive and without evidence of disease 105 months after therapy. The HPV18-positive sample was interpreted as an AC, staged T2N0M0. The patient was treated with a TLE. Nine months after surgery the tumor recurred. A RND was performed and the patient is alive and without evidence of disease 215 months after salvage surgery. Interestingly, two of the four cases, which live without evidence of disease were hrHPV-positive compared with none of the five patients who died of their disease or are alive with disease. The patient with the p16^INK4A^ positive AC presented with an early-stage glottic tumor and was successfully treated by endolaryngeal CO_2_-laser resection. This patient died of a second primary non-small cell lung cancer 19 months after surgery without evidence of recurrence of the LNEC.

## Discussion

### Laryngeal neuroendocrine carcinoma

The WHO classification divides LNEC in typical and atypical carcinoid tumors (TC, AC, respectively), and SCNEC [8]. Several studies consider LCNEC a separate category [20]. Significant progress has been made in determining an appropriate treatment strategy for the different histological tumor subtypes. A prognostic marker that allows for better pre-treatment assessment of response to therapy and clinical outcome could further improve on these efforts. Despite the small number of cases, the results from this study imply that the involvement of hrHPV in LNEC might be associated with a better response to therapy and prognosis for these tumors.

### Human papillomavirus detection

HPV consists of a family of over 120 viruses [21]. Not all of these viruses have the same oncologic potential. About a dozen viruses have been designated as ‘high-risk’ including the most common HPV16 and HPV18. The reported association between HPV and oropharyngeal cancer is also based largely on these two high-risk viruses [22, 23]. There is large variance in the reported prevalence of HPV in head and neck carcinomas. This might be caused by the different sensitivity and specificity of respective analytical methods used for detecting HPV. PCR-based detection probably overestimated the percentage of positive patients, as very few viral particles are needed to produce a positive test result [23]. Thus, PCR-positivity might represent transient not-clinically relevant low copy HPV load. In our study we used a PCR-based HPV-detection assay including serial dilution series of HPV-positive cell lines to determine high copy load. Two patients tested positive for hrHPV (HPV16 and HPV18). To evaluate the possible attributive effect of HPV16 to the malignant transformation of the laryngeal mucosa, biologically active viral infections have been associated with up-regulated protein p16^INK4A^ expression through inactivation of pRB by HPV16-E7 [24]. However, both hrHPV-positive LNEC cases were p16-negative. A possible explanation for the lack of p16^INK4A^ expression in the HPV16-positive LNEC is that hrHPV virus infection may have played a role in the carcinogenesis in an earlier stage, but the virus was inactive at the time of sampling. In agreement with this assumption, loss of the CDKNA2 gene encoding p16 is found in precursor fields (reviewed by Leemans [25]). On the other hand, despite the high association between HPV16 positivity and p16^INK4A^ expression in tonsillar carcinomas [19], in various studies a disagreement was observed in the detection of HPV DNA and p16^INK4A^ [26, 27]. These observations suggest that p16^INK4A^ cannot serve as a reliable surrogate marker in HNSCC and might support our findings that our HPV-positive samples are p16^INK4A^ -negative. The prevalence of tumors in our study that appear to be associated with HPV (2 out of 10) is in line with the estimated worldwide prevalence of HPV-induced HNSCC [28].

### p53 expression

The possibility of HPV-induced malignant transformation was further explored by p53 expression. Mutation in p53 plays an important role in tobacco- and alcohol-related cancers [29]. It results in protein overexpression of p53, accumulation of genetic damage, and eventually, uncontrolled cell proliferation. While p53 expression can also be elevated in HPV-induced cancer, this is thought to be a physiological response to increased cell proliferation [22]. The retained expression of wild-type p53 is thought to be one of the reasons why HPV-induced cancers have a relatively good prognosis. It is hypothesized that the intact p53-mediated apoptotic response to chemo- or radiotherapy-induced stress is responsible for this mechanism [5]. No p53 overexpression was found for the HPV-positive samples in this our suggesting that an alcohol- or tobacco-related cause for these cancers is less likely.

### Ki-67 expression

Further information on tumor proliferation was obtained through measurement of the expression of Ki-67. The fraction of Ki-67-positive tumor cells (the Ki-67 labeling index) is often correlated with the clinical course of the disease [30]. HPV-induced cancers are known to be associated with high levels of Ki-67 [31]. Indeed, both HPV-positive samples in our series had a high Ki-67 labeling index, whereas this was only true for one out of the eight HPV-negative cases.

### HPV status and prognosis

Due to the small sample size, no firm conclusions can be drawn regarding the relationship between HPV positivity and treatment outcome. However, it is remarkable that the two hrHPV DNA-positive cases had the longest survival in this series. Both patients were without evidence of disease 105 and 215 months after initial treatment despite having bad prognostic subtypes. Despite the fact that the patient with the p16^INK4A^-positive sample died of another malignancy without evidence of recurrence of the LNEC, p16^INK4A^ positivity was also associated with a better treatment response of the LNEC. In this study four patients remain alive with no evidence of disease: one with a TC, two with an AC, and one with a LNEC. TC are known to have a good prognosis, but the other three patients survived despite unfavorable tumor subtypes. Remarkably, two of these three patients were HPV positive. This finding is in line with previous observations in HPV-positive head and neck cancer [5–7, 32, 33]. However, in laryngeal cancer in particular, these favorable prognostic features of HPV-positive tumors have not been detected so far [34, 35]. No strong conclusions can be drawn from our series due to the small sample size and differences pertaining to tumor stage on presentation and applied treatment modality. Both HPV-positive patients were primarily treated with aggressive surgery, while other LNEC patients were subjected to various other treatment modalities including minimally invasive endoscopic surgery, radiotherapy, and chemo-radiotherapy. The exact mechanism behind the better response rate of HPV-positive head and neck tumors to (chemo) radiotherapy is still unknown. However, genetic differences, like p53 mutation or EGFR expression level, between HPV negative and positive tumors have been proposed [36, 37].

## Conclusions

We describe the detection of hrHPV in LNEC for the first time. Two out of ten tissue samples of LNEC tested positive for hrHPV. These tumors had a high Ki-67 labeling index and very good prognosis. Despite the small number of cases, the results suggest that the involvement of hrHPV in LNEC might be associated with a better response to therapy and prognosis for these tumors. The detection of hrHPV might act as a potential marker in determining individual optimal treatment strategy. An independent larger series of LNEC is needed to confirm our findings.
